# Decreased Breg/Th17 Ratio Improved the Prognosis of Patients with Ulcerative Colitis

**DOI:** 10.1155/2018/5760849

**Published:** 2018-03-22

**Authors:** Xue Bing, Liang Linlang, Chen Keyan

**Affiliations:** ^1^Department of Endocrinology, General Hospital of Shenyang Military Area Command, No. 83 Wenhua Road, Shenyang, Liaoning Province 110016, China; ^2^Department of Laboratory Animal Science, China Medical University, No. 77 Puhe Road, Shenyang North New Area, Shenyang, Liaoning Province 110122, China

## Abstract

**Objective:**

To investigate the effects of regulatory B (Breg) cells and T helper 17 (Th17) cells on pathogenesis of ulcerative colitis, explore the clinical significance of Breg/Th17 ratio on the prognosis of ulcerative colitis, and provide the theoretical basis for the targeted therapy, diagnosis, and prognosis of the disease.

**Methods:**

Peripheral blood and colonic mucosa were collected from patients with ulcerative colitis. Hematoxylin-eosin staining was used to observe the pathological changes of colonic mucosa. Flow cytometry was utilized to analyze the percentages of Breg cells and Th17 cells. Real-time fluorescent quantitative polymerase chain reaction and immunohistochemistry were applied to determine the expression of Breg cells-related cytokines IL-10 and Th17 cell transcription factor ROR*γ*T. Enzyme-linked immunosorbent assay was employed to detect serum IL-10 and IL-17 levels.

**Results:**

The colonic mucosa of ulcerative colitis patients presented massive inflammatory cell infiltration and hemorrhagic necrosis. The number of Breg cells and Th17 cells, the gene expressions of IL-10 and ROR*γ*T, and serum levels of IL-10 and IL-17 all increased in peripheral blood. Compared with nonremission group, the remission group showed that the percentage of Breg cells reduced, the percentage of Th17 cells increased, and thus the B10/Th17 ratio was significantly decreased in peripheral blood. In addition, serum IL-10 levels diminished, IL-17 levels increased, and thus IL-10/IL-17 ratio was remarkably reduced in remission group. B10/Th17 ratio and IL-10/IL-17 ratio were positively correlated with the severity of disease.

**Conclusions:**

Breg and Th17 cells participate in the occurrence and development of ulcerative colitis. B10/Th17 ratio and IL-10/IL-17 ratio can be used as prognostic markers for ulcerative colitis. This provides a theoretical basis for design of targeted treatment and prognosis assessment of the disease.

## 1. Introduction

Ulcerative colitis is a recurrent chronic immune disease. After the drug-induced remission, the symptoms and pathological examinations of the patients can be relieved to a great extent, but the condition still easily occurs again. Currently, in the treatment of ulcerative colitis, the induced remission can reduce the risk of ulcerative colitis-induced cancer [1]. In recent years, many serum [2, 3] and fecal [4, 5] markers have been applied to evaluate the prognosis of ulcerative colitis. However, regions of the intestinal mucosal inflammation, disease status, and drug administration can cause changes in these indicators [6, 7]. Thus, reliable biological markers are urgently needed for the diagnosis of ulcerative colitis and the evaluation of the disease prognosis.

B cells regulate immune responses by producing antigen-specific antibodies. However, specific B cell subsets also have the ability to modulate immune responses. Regulatory B cell (Breg), first proposed by Mizoguchi and Bhan [8], is a B cell subset that plays negative regulatory functions that inhibit immune response. Breg cells mainly affect the regulatory T cell activation and the production of immune response by producing interleukin-10 (IL-10), reducing the expression of MHC-II molecules on cell surface and downregulating tumor necrosis factor [9]. Breg exerts its immunomodulatory effects by secreting IL-10, known primarily as B10 cell [10]. T helper 17 (Th17) cell is a new type of effector T cell subset, and, like Th1 and Th2 subpopulation cells, it is differentiated from the progenitor T cells and strongly associated with autoimmune diseases and inflammatory responses [11]. Interleukin-17 (IL-17) is the major effector molecule secreted by Th17 cells. By secreting IL-17, Th17 mobilizes, recruits, and activates neutrophils and macrophages, mediates inflammatory cells to local invasion and tissue damage, and induces inflammatory responses [12].

This study further investigated the effects of Breg cells and Th17 cells on ulcerative colitis by determining peripheral blood Breg cells, Th17 cells, and related molecules in patients with ulcerative colitis and provided theoretical basis for targeted therapy and prognosis of ulcerative colitis.

## 2. Materials and Methods

### 2.1. Subjects and Group Assignment

Thirty-three patients with ulcerative colitis at the age between 23 and 59 years old were collected from the Department of Gastroenterology of the General Hospital of Shenyang Military of China from July 2016 to April 2017. These patients suffered from pus and blood in stool more than 3 times. The patients had not been treated with hormones, salicylic acid, other immunosuppressive agents, and antibacterial agents within 2 weeks. Thirty-four patients in remission orally took hormones, salicylic acid, or other immunosuppressive agents to achieve remission of clinical symptoms. Thirty-five patients in nonremission orally took hormones, salicylic acid, or other immunosuppressive agents to achieve remission of clinical symptoms. Thirty-five healthy controls in the control group were recruited from Hospital Physical Examination Center. Ulcerative colitis patients with one or more of the following conditions were excluded from this study: tumor and immune disease. The protocols were approved by the Ethics Committee of the General Hospital of Shenyang Military. Patients signed informed consent.

### 2.2. Sample Preparation

Venous blood (5 ml) was collected from healthy controls, ulcerative colitis patients, remission patients, and nonremission patients. 4 ml of the venous blood was used for isolation of mononuclear cells and 1 ml for serum isolation. All samples were stored at −80°C for enzyme-linked immunosorbent assay (ELISA). The colonic mucosa of ulcerative colitis patients, remission patients and nonremission patients was obtained by colonoscopy, fixed in formaldehyde, and stored at −80°C. Normal colons were harvested by colon surgery (except tumor patients).

### 2.3. Hematoxylin-Eosin Staining

The colons were fixed in 10% formaldehyde, dehydrated, embedded in wax, and sliced into sections. The sections were dewaxed, hydrated, stained with hematoxylin for 5 minutes, washed with running water for 5 minutes, differentiated with 1% hydrochloric acid in ethanol for 1–3 seconds, washed with running water for 30 seconds, stained with 0.5% eosin for 1–3 minutes, washed with distilled water for 5 seconds, dehydrated, permeabilized, and mounted with neutral resin. The changes in tissue structure were observed under the light microscope.

### 2.4. Isolation of Peripheral Blood Mononuclear Cells

Anticoagulated blood (4 ml) was collected from each group, diluted in an equal volume of PBS, lysed with an equal volume of erythrocyte lysate on the ice for 5 minutes, and centrifuged at 1000 rpm/min for 5 minutes. After removal of the supernatant, 5 ml PBS was added and centrifuged at 800 rpm/min for 5 minutes. The supernatant was discarded. RPMI-1640 cell culture medium was added for resuspension and cells were quantified.

### 2.5. Fluorescent Labeling of Peripheral Blood CD24^hi^CD27^+^CD38^hi^IL-10^+^(B10) Cells and Th17 Cells

Cells (1 × 10^6^) were placed in 12-well plates, and RPMI-1640 medium was added to a total of 1 ml. LPS (Sigma, L2880) 10 *μ*g/ml was added and incubated in 5% CO_2_ incubator at 37°C for 24 hours. 19 hours later, cells in each well were stimulated with PMA (Sigma, P1585), ionomycin (Sigma, I-0634), and Brefeldin A (BD, 555029) to reach the final concentrations as 10 ng/ml, 0.5 *μ*g/ml, and 1 *μ*l/ml, respectively. Afterwards, cells were centrifuged at 1500 rpm for 5 minutes in EP tubes. After removal of the supernatant, cells were washed twice with PBS 1 ml. The supernatant was discarded. PBS 100 ul was added, and then FITC-anti-CD24 (BD, 555427), APC-anti-CD27 (BD, 561400), PerCP-CY5.5-anti-CD38 (BD, 561106), and FITC-anti-CD4 (BD, 555427) were added and incubated at room temperature in the dark for 30 minutes. Samples were washed with cell staining buffer, incubated with 500 *μ*l fixation/permeabilization solution (BD, 555028) at room temperature in the dark for 30 minutes, washed with 1 × BD perm/wash buffer, incubated with PE-anti-IL-10 (BD, 554498) and PE-anti-IL-17 (BD, 561400) at room temperature in the dark for 45 minutes, washed with 1 × BD perm/wash buffer, and resuspended with 300 *μ*l flow washing liquid. Results were analyzed using flow cytometry.

### 2.6. Real-Time Fluorescent Quantitative Polymerase Chain Reaction (qRT-PCR)

The colons after trituration and lymphocytes after 24 hours of stimulation were placed in EP tubes. Other procedures were conducted in accordance with the instruction of Trizol reagent (15596018; Invitrogen). After precipitation and drying, 50 *μ*l of DEPC-treated water was added, and 1.2% MOPS-denaturing formaldehyde gel electrophoresis was conducted. In accordance with RevertAid™ First Strand cDNA Synthesis Kit (K1621, Thermo), RNA was reverse transcribed into first strand cDNA. Taking GAPDH as reference gene, IL-10 and ROR*γ*T were quantitatively analyzed with SYBR Green PCR kit (204054, Qiagen). The relative amount of each target gene/reference gene was calculated. According to the sequences of IL-10 and ROR*γ*T in Genbank, DNAman software was utilized for primer design. Primers were synthesized by Sangon Biotech (Shanghai) Co., Ltd., China. Primer sequences are as follows:

  IL-10-F: GACTCTATAGACTCTAGG
  R: CATCAACTACATAGAAGC
  ROR*γ*T-F: AACAACTTGGCCAAGGCA
  R: GGGACAGGGCCCAGACAG
  GAPDH-F: GCTCATTTGCAGGGGGGA
  R: CACCACCAACTGCTTAGC

### 2.7. Immunohistochemistry

Paraffin sections were dewaxed, hydrated, and blocked with 3% H_2_O_2_ for 15 minutes to inactivate endogenous peroxidase. Antigens were retrieved with 10 mM sodium citrate buffer. After three washes with PBS, samples were blocked with goat serum for 30 minutes and incubated with anti-IL-10 (Abcam, ab134742) and anti-IL-17 (Abcam, ab92486) at 4°C overnight. After three washes with PBS, secondary antibody was added and incubated at 37°C for 2 hours. Nuclei were counterstained with hematoxylin. Samples were visualized with 3,3′-diaminobenzidine, mounted with neutral resin, and observed under the light microscope.

### 2.8. ELISA

Serum was collected from healthy controls, ulcerative colitis patients, remission patients, and nonremission patients. In accordance with the instructions of IL-10 (CCC, SEA056Hu) and IL-17 (CCC, SEA688Hu) reagents, optical density (OD) values were measured at 450 nm using a microplate reader. The standard curves used OD values as *y*-axis, and standard sample concentrations as *x*-axis. Curve equation and *r* value were calculated, and sample concentrations were measured.

### 2.9. Correlation Analysis between Percentages of B10 and Th17 Cells in Peripheral Blood and the Severity of the Disease

According to Mayo scoring system [13], the severity of the disease was assessed in all patients as follows: Mayo scoring ≤ 2 points, remission; 3–5 points, mild; 6–10 points, moderate; 11-12 points, severe (Table 1). Correlation analysis in peripheral blood B10 cells and Th17 cells as well as B10/Th17 ratio was carried out in patients in remission, with mild, moderate, and severe ulcerative colitis.

### 2.10. Statistical Analysis

All data were analyzed using SPSS 19.0 software. Data in each group were compared and analyzed using group *t*-test, one-way analysis of variance, and Spearman's correlation. The data were expressed as mean ± standard deviation. A value of *P* < 0.05 was considered statistically significant.

## 3. Results

### 3.1. Intestinal Morphological Changes after Ulcerative Colitis

**Figure 1:**
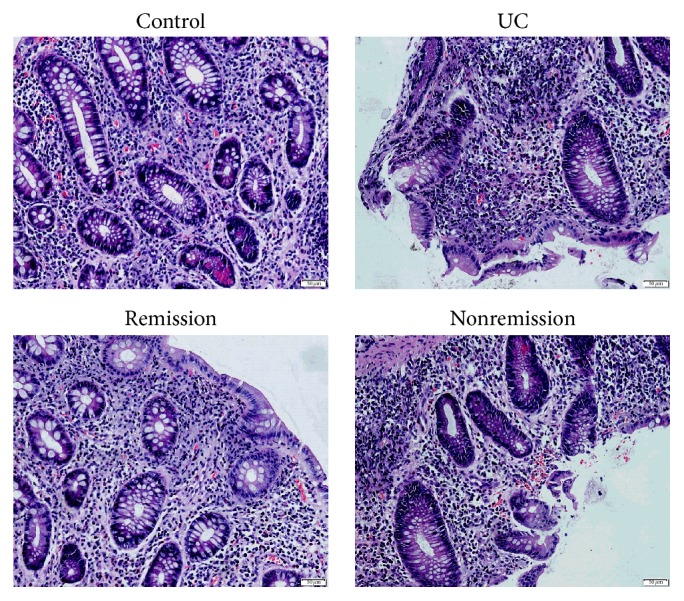
Intestinal morphological changes after ulcerative colitis by HE staining; UC group showed a lot of inflammatory cell infiltration. In remission, a small number of inflammatory cell infiltrations and bleeding were visible in colonic mucosa.

In the control group, colonic mucosa was normal and cells were regularly distributed (Figure 1). In the ulcerative colitis group, colonic mucosa presented bleeding, edema, a large number of inflammatory cell infiltrations, intestinal epithelial cell degeneration, and necrosis. In patients in remission, a small number of inflammatory cell infiltrations and bleeding were visible in colonic mucosa; edema was remarkably lessened compared with ulcerative colitis patients. A large number of inflammatory cell infiltrations, intestinal epithelial cell degeneration, and necrosis were observed in the nonremission group.

### 3.2. B10 Cell and Th17 Cell Counts in Ulcerative Colitis Patients and Healthy Subjects

**Figure 2:**
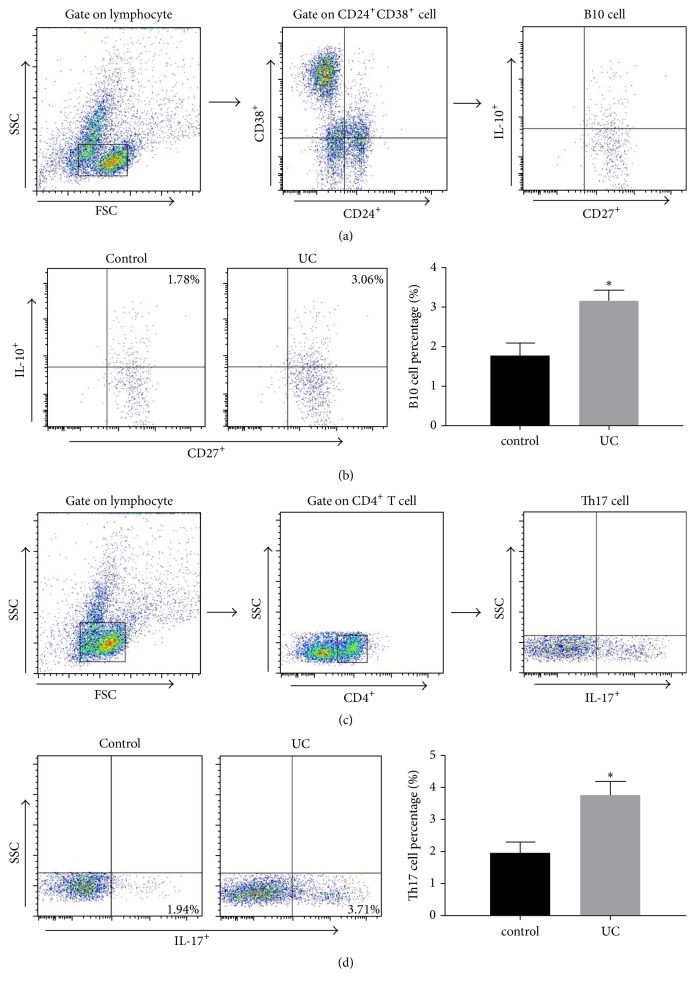
B10 cell and Th17 cell counts in ulcerative colitis patients and healthy subjects. (a-b) Gating criteria to define the CD24^+^CD27^+^CD38^+^IL-10^+^ Breg cell population. Different cell subsets were distinguished according to different cell labels. (c-d) The percentage of CD4^+^IL-17^+^ cells in peripheral blood. Compared with control group, ^*∗*^*P* < 0.05.

To study the B10 cell and Th17 cell counts in ulcerative colitis patients and healthy controls, cell counting was performed with flow cytometry. Our results revealed that CD24^hi^CD27^+^CD38^hi^IL-10^+^ (B10) percentage in peripheral blood was significantly higher in the ulcerative colitis patients than in controls (*P* < 0.05) (Figures 2(a)-2(b)). Moreover, the percentage of CD4^+^IL-17^+^ cells was significantly higher in the ulcerative colitis patients than in controls (*P* < 0.05) (Figures 2(c)-2(d)). These data suggested that the percentages of B10 cells and Th17 cells increased in peripheral blood of ulcerative colitis patients, which were possibly associated with the onset of disease.

### 3.3. The Expressions of IL-10 and ROR*γ*T in Ulcerative Colitis Patients and Healthy Subjects

**Figure 3:**
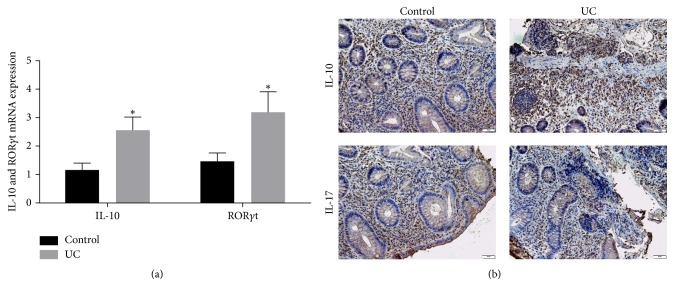
The expression of IL-10 and ROR*γ*T in ulcerative colitis patients and healthy subjects was upregulated. (a) The mRNA expression levels of IL-10 and ROR*γ*T were determined with qRT-PCR. (b) IL-10 and IL-17 expression by immunohistochemistry. Compared with control group, ^*∗*^*P* < 0.05.

Based on the findings about the association between Breg and Th17 cells with pathogenesis of ulcerative colitis, the mRNA expression levels of IL-10 and ROR*γ*T were determined with qRT-PCR. Our results showed that, compared with the healthy controls, IL-10 mRNA expression was significantly increased in ulcerative colitis patients (*P* < 0.05) (Figure 3(a)). Compared with healthy controls, ROR*γ*T mRNA expression was also significantly increased in the UC patients (*P* < 0.05).

Immunohistochemistry results showed that IL-10 and IL-17 were also significantly increased in the UC patients (Figure 3(b)). In line with the above findings, these results suggested that the expressions of IL-10 and Th17 cell-specific transcription factors were upregulated in ulcerative colitis patients.

### 3.4. Serum Levels of IL-10 and IL-17 in Ulcerative Colitis Patients and Healthy Subjects

**Figure 4:**
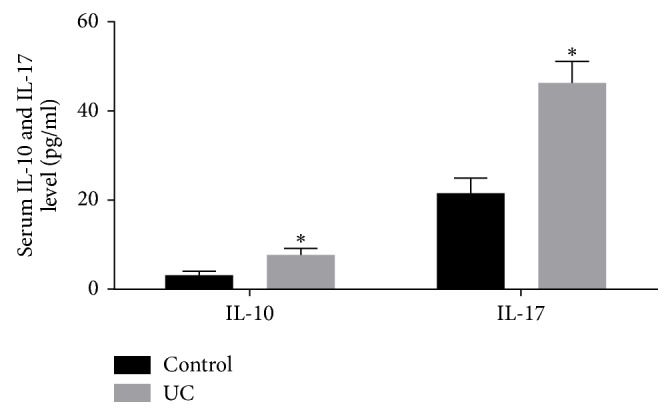
Serum levels of IL-10 and IL-17 in ulcerative colitis patients and healthy subjects were detected with ELISA. IL-10 and IL-17 levels were significantly higher, compared with control group, ^*∗*^*P* < 0.05.

Serums IL-10 and IL-17 in ulcerative colitis patients and healthy subjects were detected by ELISA. Our results demonstrated that serum IL-10 levels were significantly higher in ulcerative colitis patients than in controls (*P* < 0.05) (Figure 4). Similar results were obtained for the detection of serum IL-17 levels in the ulcerative colitis patients and controls (*P* < 0.05) (Figure 4). These findings suggested that, in line with the alterations in Breg and Th17 cells, the serum levels of IL-10 and IL-17 were increased in the ulcerative colitis patients.

### 3.5. Cell Counts and Cytokine Levels in Ulcerative Colitis Patients in Remission and Nonremission after Treatments

**Figure 5:**
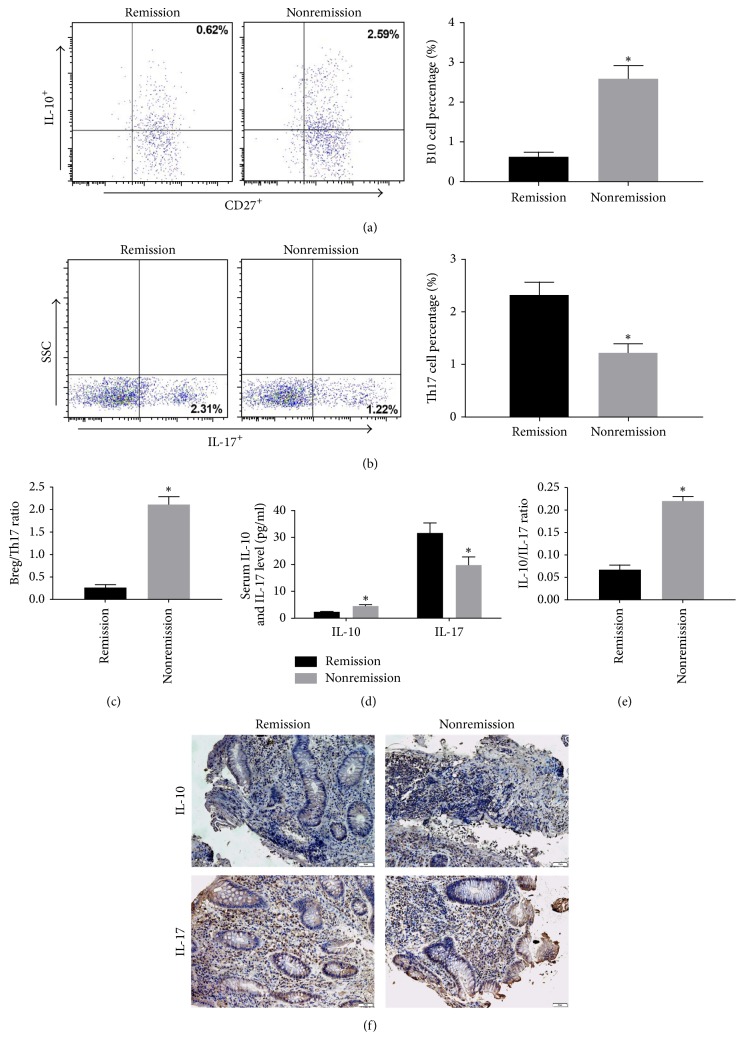
The differences in the cell counts and related cytokine levels between ulcerative colitis patients in remission and nonremission after treatment were analyzed with flow cytometry and ELISA. (a) Gating criteria to define the CD24^+^CD27^+^CD38^+^IL-10^+^ Breg cell population in the remission group and nonremission group. (b) The percentage of Th17 cells in the remission group and nonremission. (c) B10/Th17 ratio in the remission group and nonremission group. (d) Serum IL-10 levels and IL-17 levels were detected with ELISA. (e) The IL-10/IL-17 ratio in the remission group and nonremission group. (f) IL-10 and IL-17 expression by immunohistochemistry in in the remission group and nonremission group. Compared with remission group, ^*∗*^*P* < 0.05.

The differences in the cell counts and related cytokine levels between ulcerative colitis patients in remission and nonremission after treatment were next analyzed and compared. Flow cytometry analysis showed that the number of B10 cells significantly decreased in peripheral blood of ulcerative colitis patients in remission (*P* < 0.05, versus nonremission group) (Figure 5(a)); the proportion of Th17 cells significantly increased (*P* < 0.05, versus nonremission group) (Figure 5(b)). Accordingly, B10/Th17 ratio in the remission group was significantly lower than in the nonremission group (Figure 5(c)). The serum levels of IL-10 and IL-17 were also determined and compared between the remission and nonremission groups. Our results showed that serum IL-10 levels significantly diminished in the remission group (*P* < 0.05, versus nonremission group) (Figure 5(d)). However, IL-17 levels significantly increased (*P* < 0.05, versus nonremission group) (Figure 5(d)). The IL-10/IL-17 ratio was significantly reduced in the remission group compared with the nonremission group (*P* < 0.05, versus nonremission group) (Figure 5(e)). The same results were also obtained by immunohistochemistry (Figure 5(f)). These results confirmed that the changes in the cell counts and related cytokine levels induced by drug treatment would contribute to the disease remission in ulcerative colitis patients.

### 3.6. Correlation of Cell Counts and Cytokine Levels with Disease Development in Ulcerative Colitis Patients

**Figure 6:**
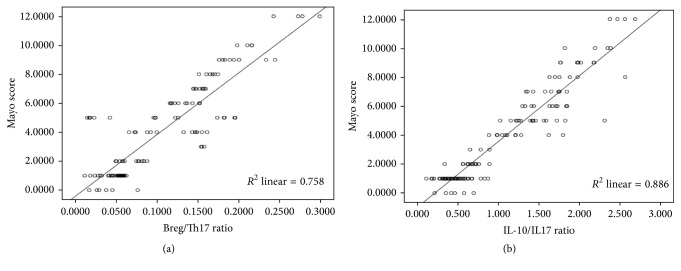
Correlation of cell counts and cytokine levels with Mayo scoring in ulcerative colitis patients, B10/Th17 ratio, and Mayo scoring were positively correlated; serum IL-10/IL-17 ratio and Mayo scoring were positively correlated. (a) Peripheral blood B10/Th17 ratio was positively correlated with Mayo in ulcerative colitis patients (*r* = 0.758, *P* < 0.05). (b) Serum IL-10/IL-17 ratio was positively correlated with Mayo in ulcerative colitis patients (*r* = 0.886, *P* < 0.05).

We analyzed the correlation of peripheral blood B10 cells, Th17 cells, and their related factors with Mayo scoring. Our results found that peripheral blood B10/Th17 ratio was positively correlated with Mayo in ulcerative colitis patients (*r* = 0.758, *P* < 0.05) (Figure 6(a)). Serum IL-10/IL-17 ratio was also positively correlated with Mayo (*r* = 0.886, *P* < 0.05) (Figure 6(b)). These data suggested that a high B10/Th17 or IL-10/IL-17 ratio indicated severe patient's condition, whereas a low B10/Th17 or IL-10/IL-17 ratio suggested good prognosis.

## 4. Discussion

The occurrence of ulcerative colitis is associated with heredity, immunity, infection, and environmental factors. The intestinal immune system is activated under the participation of environmental factors and intestinal flora [14, 15]. In the presence of persistent antigenic stimuli and (or) immune dysregulation, inflammatory cascade magnifies and local inflammatory mediators injure tissues, thereby resulting in the occurrence of ulcerative colitis. In this study, the number of peripheral blood Breg and Th17 cells increased in ulcerative colitis patients, which were possibly associated with the occurrence and development of disease. In remission, the percentage of B10 cells diminished, but the percentage of Th17 cells obviously increased in peripheral blood of ulcerative colitis patients. B10/Th17 ratio was significantly lower in the remission group than in the nonremission group. IL-10 levels remarkably reduced, but IL-17 levels noticeably increased in remission. IL-10/IL-17 ratio was significantly declined. These results indicated that, after drug treatment, the counts of B10 cells and Th17 cells and the expression of related cytokines contributed to the remission of ulcerative colitis.

At present, the negative immune regulatory function of Breg cells plays an important role in autoimmune response [16]. Harris [17] suggested that, in inflammatory conditions, B cells are similar to CD4^+^ T cells and produce cytokines to fight against inflammation. Wolf et al. [18] found that the induction of experimental autoimmune encephalomyelitis in B cell-deficient mice aggravated the severity of the disease, but the same induction in wild-type mice could mitigate the symptoms. Xiao et al. [19] found that, in models of mutation of Fas gene, Breg cells (B10) that secreted IL-10 had abnormal function; mice presented severe lupus, and the pathogenic antibodies of lupus were also significantly elevated. It is thus clear that Breg cells from B cells have a negative immune regulation mechanism, and B cells play an immune regulatory role in autoimmune diseases.

Th17 cell is a new type of CD4 effect T cell that is different from the traditional Th1 and Th2 cells. It plays an important role in the pathogenesis of inflammatory diseases and autoimmune diseases [20]. IL-17 is the major effector molecule secreted by Th17 cells. By secreting IL-17, Th17 mobilizes, recruits, and activates neutrophils and macrophages, mediates inflammatory cells to local invasion and tissue damage, and induces inflammatory responses [21]. Recent data has demonstrated that biologics neutralizing IL-17 (ixekizumab and secukinumab) or its receptor (brodalumab) are highly effective with a positive safety profile in treating moderate to severe psoriasis [22]. In this study, we found that abnormal expression of Breg and Th17 cells in peripheral blood of UC patients may lead to immune imbalance, so that the effector type CD4^+^ T cells migrate from the circulatory system to the local inflammatory site of the intestinal tract, enrichment in the inflammatory site, leading to digestion mucosa in a highly active state, induced by the intestinal response, resulting in self-antigen intolerance and increased release of damage to cytokines, leading to mucosal injury.

In summary, Breg cells and Th17 cells participate in the occurrence and development of ulcerative colitis. B10/Th17 ratio and IL-10/IL-17 ratio can be used as prognostic markers of ulcerative colitis. This provides a theoretical basis for targeted therapy of clinical drugs and disease prognosis.

## Figures and Tables

**Table 1:** Mayo score.

Terms	≤2 points	3–5 points	6–10 points	11-12 points
Stool frequency	Normal	1-2 stools/day more than normal	3-4 stools/day more than normal	>4 stools/day more than normal
Rectal bleeding	None	Visible blood with stool less than half the time	Visible blood with stool half of the time or more	Passing blood alone
Mucosal appearance at endoscopy	Normal or inactive disease	Mild disease (erythema, decreased vascular pattern, mild friability)	Moderate disease (marked erythema, absent vascular pattern, friability, erosions)	Severe disease (spontaneous bleeding, ulceration)
Physician rating of disease activity	Normal	Mild	Moderate	Severe

*Notes*. Mayo scoring ≤ 2 points: remission; 3–5 points: mild; 6–10 points: moderate; 11-12 points: severe.
